# An unusual location of a cavernous hemangioma: a case report

**DOI:** 10.11604/pamj.2021.39.29.28492

**Published:** 2021-05-11

**Authors:** Zahra Sayad, Bouchra Dani, Salma Benazzou, Malik Boulaadas

**Affiliations:** 1 Department of Oral and Maxillofacial Surgery, Ibn Sina University Hospital Center, Rabat, Morocco

**Keywords:** Cavernous, hemangioma, diagnostic, treatment, case report

## Abstract

Hemangiomas are benign vascular tumors that most often affect the skin, mucous membranes, subcutaneous tissues, bone and on rare occasions muscles. In the head and neck region, the masseter and trapezius muscles are most often affected; the temporalis muscle involvement is extremely rare. It is a childhood pathology that rarely occurs in adults. We report a case of a cavernous hemangioma in a 37-year-old female. Through this case and in the light of literature we focus on the clinicopathological aspects of this tumor and the rarity of this location.

## Introduction

Intramuscular hemangiomas are benign vascular neoplasms that represent less than 1% of all hemangiomas and which are often localized in the trunk and extremities. There are three types of hemangioma depending on the size of the affected vessel: capillary hemangioma, cavernous hemangioma, and compound hemangioma [1]. We report a case of a rare location of a cavernous hemangioma in the temporalis muscle with an extension to the infra temporal fossa.

## Patient and observation

A 37-year-old female, without significant personal or family medical history. The patient presents a swelling of the left temporal fossa that has been evolving for 5 years by gradually increasing in volume leading to a facial asymmetry. The physical examination revealed a soft, painless, non-pulsatile mass (4x2.5cm in size), without thrill, not increasing volume in the declivity position, fixed to the deep plane, mobile to the skin, without inflammatory signs or lymphadenopathy.

**Figure 1:**
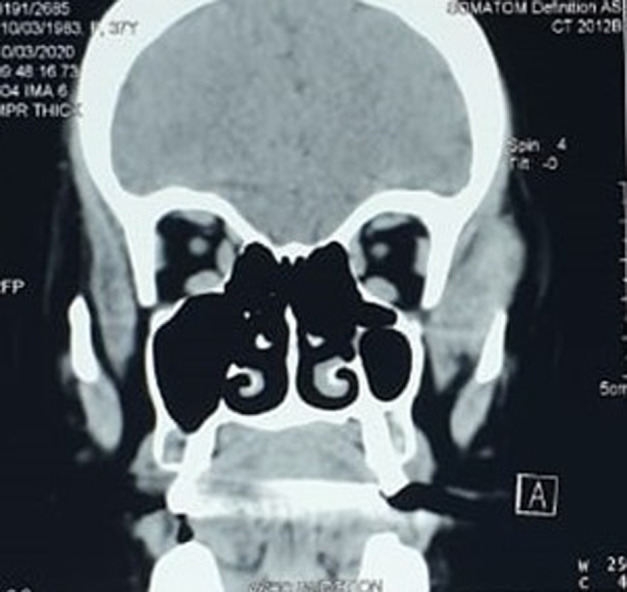
computed tomography scan on the coronal plane shows the mass in the left temporal region with no erosion of the bone

**Figure 2:**
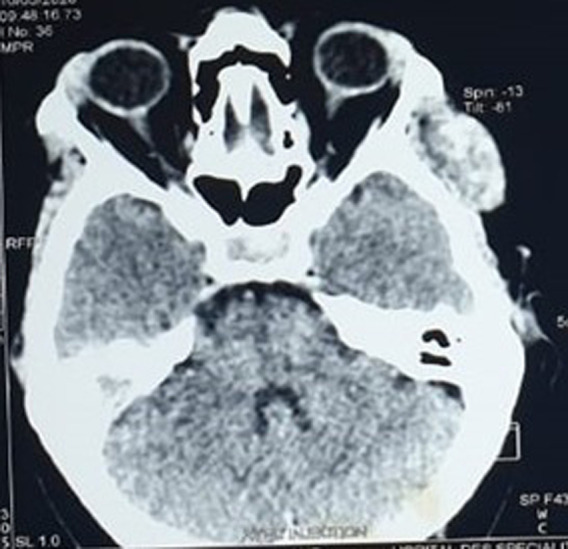
computed tomography scan on the axial plane shows a homogeneous contrast enhancement of the mass

Contrast-enhanced computed tomography was performed, showing a temporalis muscle mass that extends to the infra-temporal fossa, measuring 33x17x39mm, isodense, unencapsulated, with polylobed contours, that enhanced after contrast injection and respected the subcutaneous fat (Figure 1, Figure 2).

**Figure 3:**
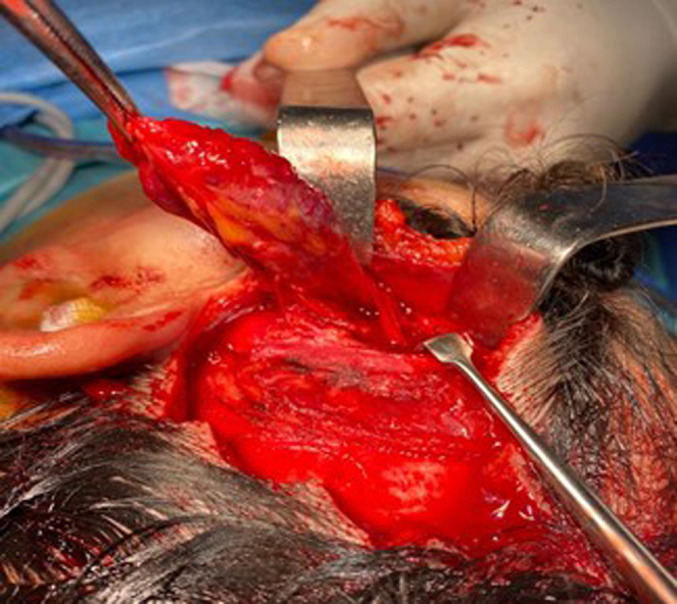
intraoperative view of the hemangioma approached through hemi coronal flap

The patient underwent surgery, where we performed a hemi-coronal incision. The mass was found in the temporalis muscle and was excised completely without damaging the frontalis branch of the facial nerve. The operating follow-ups were simple, with excellent functional and aesthetic results (Figure 3).

**Figure 4:**
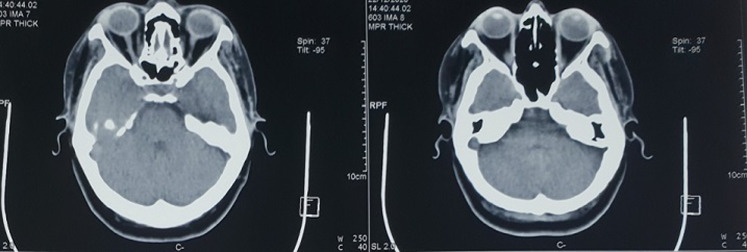
control computed tomography scan performed two years later was normal without mass

Macroscopic mass examination showed a smooth, nodular tumor tissue measuring 5x2.5x1cm, with a purplish-brownish appearance. The histological study found a benign proliferation of dilated blood vessels with variable shape and size often cavernous that were trapped between muscle fibers; they are bordered by a well differentiated endothelium and supported by fibrous interstitial tissue in favor of cavernous hemangioma. Over a two-year period, no signs of recurrence were detected (Figure 4).

## Discussion

Hemangiomas are benign tumors characterized by abnormal proliferation of blood vessels. Their intramuscular locations represent less than 1% of all hemangiomas. Only 14% are located at the head and neck region. Masseter (36%), trapezius (24%) and sternocleidomastoid muscles are the most affected, while temporal muscle involvement remains extremely rare. To our knowledge, only 29 cases have been reported in the literature. A slight female predominance was described with an average age of 33 years [1, 2].

The etiopathogenesis of these tumors are unknown, yet there are several theories: congenital, hormonal factors and trauma. There are three types depending on the size of the vessel involved: capillary (small vessels) most common with an estimated incidence of 68%, Cavernous (26%) (Large vessels) followed by compound types (6%). Cavernous hemangioma is most frequently found in the temporal muscle [2, 3]. Clinically, they appear as a slow growing mass, mobile, painless, without pulsation, and without staining of the skin. The differential diagnosis includes lipoma, neurofibroma, dermoid cyst, enlarged lymph nodes, soft-tissue sarcoma, myositis ossificans and temporal arteritis [1, 4].

Imaging help to make a positive diagnosis and guiding the management of this tumor. Computed tomography is useful for defining the form, size, eventual bone damage, and infiltration of the surrounding tissues. However, magnetic resonance imaging (MRI) is the method of choice in defining the vascular nature of the tumor and providing further information on the exact extent of the tumor. Hemangiomas are generally isointense to muscle on T1-weighted images and hyperintense on T2-weighted images. Arteriography allows to identify the feeding vessels of the mass for a preoperative embolization if necessary [1, 3, 5].

Several therapeutic modalities are available ranging from simple observation, injection of sclerosing agents, corticosteroid treatment, radiotherapy, embolization (especially in preoperative) to reduce intraoperative bleeding, arriving at complete surgery excision which remains the method of choice in the definitive treatment of this tumor. In some cases of voluminous tumor, injection of sclerosing agents, corticosteroid treatment and radiotherapy, can be indicated as alternatives or adjuvants to surgery [6, 7]. In our case, under general anesthesia, hemi-coronal incision is used to control the region and the careful surgical dissection allows to prevent injury of the temporal and auricular branches of the facial nerve. The therapeutic indications are made according to the age, the location and size of tumor, growth rate, vascularity, refractory pain, cosmetic malformations, and suspicion of malignancy [8, 9].

Local recurrence is possible after an incomplete resection with rates estimated at 28% for the compound type, 20% for the capillary and 9% for the cavernous. Close and prolonged Clinical and radiological follow up are recommended for at least 2 years to ensure immediate diagnosis of eventual local recurrence [9].

## Conclusion

Cavernous hemangioma of the temporalis muscle is a benign and rare entity. The clinical presentation is unspecific. Imaging plays a very important role in the diagnosis, especially the MRI which remains the examination method of choice. Despite the multitude of therapeutic means, surgery retains its place in the definitive treatment of cavernous hemangiomas.
